# Polyradiculonévrite inflammatoire démyélinisante chronique révélant une thrombocytémie essentielle

**DOI:** 10.11604/pamj.2014.19.101.3121

**Published:** 2014-09-29

**Authors:** Neirouz Ghannouchi Jaafoura, Amira Atig, Ahmed Bouker, Omar Alaoua, Mabrouk Khalifa, Fathi Bahri

**Affiliations:** 1 Service de Médecine Interne, CHU Farhat Hached, Sousse, Tunisie

**Keywords:** Polyradiculonévrite, thrombocytémie, affection, Polyradiculonevritis, thrombocytemia, disorder

## Abstract

La polyradiculonévrite inflammatoire démyélinisante chronique est une affection rare qui se caractérise par une neuropathie sensitivo-motrice démyélinisante, segmentaire et multifocale de mécanisme probablement dys-immunitaire et répondant à un traitement immunomodulateur. Une étiologie sous jacente est retrouvée dans près de 40% des cas et peut être infectieuse, auto-immune ou tumorales que ce soit les néoplasies solides ou les hémopathies notamment les gammapathies monoclonales. Nous rapportons une association exceptionnelle de polyradiculonévrite inflammatoire démyélinisante chronique qui révèle une thrombocytémie essentielle.

## Introduction

La thrombocytémie essentielle (TE) est un syndrome myéloprolifératif (SMP) qui expose au risque de complications vasculaires thrombotiques. Les manifestations neurologiques périphériques sont exceptionnelles. A travers cette observation, nous rapportons la survenue d'une polyradiculonévrite inflammatoire démyélinisante chronique (PIDC) révélatrice d'une TE.

## Patient et observation

Madame M B, âgée de 48 ans, sans antécédents pathologiques notables, est hospitalisée en décembre 2010 pour exploration de troubles de l’équilibre, évoluant depuis 6 mois, avec sensation de lourdeur des membres inférieurs, précédée un mois auparavant par des fourmillements et des crampes musculaires. A l'examen physique, la patiente était apyrétique, avec un état général conservé, un poids de 88 kg, une taille de 1m72 et une pression artérielle debout et couchée normale à 110/70 mmHg. L'examen neurologique objectivait une ataxie proprioceptive, une marche dandinante nécessitant une aide, des réflexes ostéo-tendineux abolis aux membres inférieurs, une sensibilité tactile et algésique conservée mais avec des erreurs du sens de positionnement du gros orteil. L'examen des paires crâniennes était normal. Il n'y avait pas par ailleurs d'adénopathies périphériques ni d'hépato ou splénomégalie. A l’éléctromyogramme (EMG), on notait une diminution des vitesses de conduction nerveuses sensitives et motrices aux membres supérieurs et leur absence au niveau des membres inférieurs ainsi qu'une absence de l'onde réflexe H et de l'onde F témoignant d'une dénervation sensitivo-motrice diffuse aux quatre membres évoquant une polyradiculonévrite démyélinisante. La ponction lombaire montre une dissociation albumino-cytologique avec une protéinorrachie à 1.26 g/l.

L'IRM cérébrale et médullaire était normale. Ainsi devant l'association de troubles sensitifs et moteurs, le résultat de l'EMG, la dissociation albumino-cytologique et l’évolution depuis six mois, le diagnostic d'une polyradiculonévrite inflammatoire démyélinisante chronique (PRNC) est retenu. L'enquête étiologique à cette PRNC s'avère négative, en effet l’étiologie toxique était éliminée par l'interrogatoire qui ne retrouvait pas de consommation de toxiques ni de prise médicamenteuses au long cours, la glycémie était à 4.6 mmol/l, la fonction rénale était normale de même que le bilan hépatique, le bilan phosphocalcique et le dosage de l'enzyme de conversion. Le bilan thyroïdien était normal, les sérologies des hépatites virales B et C et du VIH étaient négatives de même que le bilan immunologique (Facteurs antinucléaires, anticorps anti-thyroïdiens et anticorps anticytoplasme des polynucléaires neutrophiles). La recherche de néoplasie était également négative avec une TDM thoraco-abdomino-pelvienne et une écho-mammographie normales. L’électrophorèse des protéines sériques était normale avec une protidémie à 67 g/l et une gammaglobulinémie à 9.4 g/l.

A la Numération de la formule sanguine, on notait des globules blancs à 6700 éléments/ mm3, une hémoglobine à 15 g/l avec hématocrite à 46% et une thrombocytose à 882 000 éléments/ mm3 contrôlée à plusieurs reprises en l'absence de syndrome inflammatoire biologique (Vitesse de sédimentation à 6 la 1^ère^ heure, fibrinémie à 3.6 g/l). Au myélogramme, la moelle était de bonne densité cellulaire avec présence de nombreux mégacaryocytes de morphologie normale. La recherche de la mutation JAK 2 et du transcrit BCR/ABL est négative et le caryotype médullaire est normal. On complète par une biopsie ostéo-médullaire (BOM) qui montre une hyperplasie mégacaryocytaire avec des mégacaryocytes de morphologie normale, il n'y avait pas de blastes ni de signes de myélofibrose. Ainsi le diagnostic d'une thrombocytémie essentielle (TE) était retenu.

Une corticothérapie, à la dose de 1mg/ kg/ jour, était entamé en association à une réeducation fonctionnelle et un traitement anti-agrégant plaquettaire sans aucune amélioration. Ainsi un traitement par immunoglobulines intraveineuses, à la dose de 1g/kg sur 3 jours toutes les 3 semaines a été associé pendant 3 mois. L’évolution était initialement favorable avec récupération partielle du déficit moteur et de l'autonomie ainsi qu'une diminution du trouble de l’équilibre. La dose des corticoïdes était réduite progressivement arrivant à 10 mg par jour à un an de traitement. En Juin 2012, la patiente rechutait avec aggravation du déficit moteur qui s’étendait aux membres supérieurs et apparition de troubles vasomoteurs aux 4 membres. A la NFS, on notait une ascension des chiffres de plaquettes à 1 227 000 éléments/mm3 avec une hémoglobine à 14.4 g/dl, une hématocrite à 43% et des globules blancs à 8600 éléments/mm3. L'enquête étiologique refaite de nouveau était négative. La dose des corticoïdes était majorée à 1mg/kg/jour de prédnisone en association à l'azathioprine à la dose de 3 mg/kg par jour et un traitement cytoréducteur par Hydroxycarbamide (hydréa) à la dose de 15 mg/kg /jour. On ne notait pas d'amélioration de l’état clinique de la patiente qui devenait grabataire, puis elle était perdue de vue.

## Discussion

La PIDC est une neuropathie rare, d'origine vraisemblablement dys-immunitaire, d’évolution chronique ou à rechute et conduisant à un handicap moteur d'intensité variable [1]. Elle se traduit par une atteinte neurologique périphérique, à l'origine d'un déficit moteur et sensitif habituellement symétrique, à la fois distal et proximal, avec une prédominance des signes moteurs et une installation progressive sur plusieurs mois. A côté des arguments cliniques, des paramètres biologiques (liquide céphalorachidien), électromyographiques et plus ou moins histologiques sont nécessaires pour le diagnostic [2].

La distinction entre formes primitives et formes secondaires a essentiellement un impact pronostique puisque les formes secondaires semblent avoir un plus mauvais pronostic avec une évolution plus fréquente vers la chronicité [3]. De nombreuses étiologies peuvent être à l'origine des PRNC qu'elles soient infectieuses, notamment secondaire à une hépatite virale B ou C [4], auto-immunes telle que certaines connectivites [5] ou vascularites systémiques [6]. Les PIDC peuvent aussi être d'origine paranéeoplasique [7]. La survenue de neuropathies périphériques (NP) au cours des hémopathies est rare [8], de l'ordre de 7%, pouvant être révélatrices ou précéder de plusieurs années la survenue d'hémopathie. Elle peut également survenir dans l’évolution avec la difficulté de distinguer un éventuel effet des divers traitements utilisés [9]. Les hémopathies les plus rapportées en association avec des NP sont les dysglobulinémies monoclonales, POEMS syndrome et les lymphomes de hodgkin ou non hodgkinien (Navellou et al, 2011). Au cours des SMP en général et des TE en particulier, la survenue de NP est exceptionnelle.

Le diagnostic de TE, reposait chez cette patiente, sur les critères de l'OMS devant la présence d'une thrombocytose > 450 000éléments / mm^3^ à plusieurs reprises, la prolifération de la lignée mégacaryocytaire à la BOM sans anomalies de la granulopoïèse, de l’érythropoïèse, ni signes de myélofibrose, avec négativité de la recherche du transcrit BCR / ABL et en l'absence d'arguments en faveur d'une thrombocytose secondaire [10].

## Conclusion

Par cette observation nous ajoutons la TE à la liste des étiologies de PIDC qui était dans ce cas révélatrice. La survenue d'une poussée, concomitante à l'ascension des chiffres de plaquettes, renforce l'hypothèse d'un lien de causalité.
